# Good governance competencies in public health to train public health physicians

**DOI:** 10.1007/s00038-015-0702-y

**Published:** 2015-07-10

**Authors:** Chiara Bertoncello, Alessandra Buja, Andrea Silenzi, Maria Lucia Specchia, Giuseppe Franchino, Agnese Lazzari, Vincenzo Baldo, Walter Ricciardi, Gianfranco Damiani

**Affiliations:** Department of Molecular Medicine, Laboratory of Public Health, University of Padua, Via Loredan 18, 35131 Padua, Italy; Department of Public Health, Catholic University of the Sacred Heart, Rome, Italy; Neural and Biomedical Sciences, Bologna University, Bologna, Italy

**Keywords:** Competencies, Health system governance, Public health education, Public health professionals

## Abstract

**Objectives:**

This study aimed at assessing public health residents’ perceived health system governance (HSG) training needs and to define a competency framework for “good governance” to improve Public Health physicians’ curricula.

**Methods:**

A questionnaire was administered to all Italian medical residents on postgraduate courses in Hygiene and Preventive medicine. Twenty-five (78.1 %) of the 32 Italian Schools of Public Health and 299/535 residents (55.9 %) took part in this survey. The public health governance competency framework was developed from roles and responsibility at different levels of governance in the Italian Health System context.

**Results:**

The questionnaire revealed that residents felt the need for more training on all the proposed HSG-related topics. Different governance functions, strategic planning, operational planning, and operational programming were considered when defining roles and responsibilities.

**Conclusions:**

More efforts should be made to provide organic training plans tailored to the needs of local and national health system. The competencies framework for good governance could be useful for planning professional training in both the academic and the health system settings.

## Introduction

In the context of a public health system in which healthcare is a right and not a privilege, good governance ensures that the health system’s rules and institutions are beneficial to the population. At every level, the aim of public health system governance is to ensure that any action taken is always constantly focusing on providing added value for the individuals and groups being served by allocating and using the available resources to maximum benefit, and by minimizing waste (i.e. anything that does not add value to the outcome) (Gray 2013). Public health professionals should govern health systems from a population medicine perspective (Gray 2013). In 2000, the WHO introduced the concept of health sector “stewardship,” which is closely related to leadership and governance. It defined stewardship as “the careful and responsible management of the well-being of the population” (Boffin 2002). Strategic planning involves defining the principles of the health system, its objectives for the population being served, and a framework or setting by means of which to achieve these objectives, including the allocation of budgets and resources at regional or local level. The operational programming function at a given center of responsibility has to translate the strategic plan into activities, which might cover the whole range of operations involved in health care provision, i.e. organizing services and providing the staff, facilities and equipment needed to reach the assigned goals. Programs and projects are the tools used in this operational programming process.

Despite the growing body of literature on the concepts of public health governance, there is still a shortage of clear and consistent tools available to help health system stewards in defining competences to carry out the governance function with its strategic planning and operational programming function (Barbazza and Tello 2014; Brinkerhoff and Bossert 2014).

There is also quite a considerable variability in the tools used on different levels (national, regional and institutional), and there may be a diversity of approaches between operators with different levels of authority and mandates from those serving in stewardship roles (Barbazza and Tello 2014).

Although the terms “governance” and “stewardship” are increasingly being used to draw attention to a number of factors affecting the quality, efficacy, and reach of social services, no consensus has emerged on their definitions, the related frameworks, and how they apply to the health sector in particular (Savedoff and Network 2011).

With a view to assuring a future for public health systems and improving public health professional training programs, the Institute of Medicine of the US Academies has highlighted the need to enhance policy training, which should be part of structured management training programs for public health residents (Gebbie et al. 2003). Such structured management training programs need to be tailored to the functions and roles required of public health managers in each national health care system. Many competency frameworks have been developed to support public health operators, (Czabanowska et al. 2014a). Such frameworks attempt to promote high standards in professional public health practice and serve as a guide to design public health curricula. Although the importance of policies has long been recognized, training is rarely provided on how to analyze, develop, and implement policies (Oni et al. 2010).

Bjegovic-Mikanovic et al. (2013) claim in their article that public health education in Europe is wholly inadequate in preparing workers for the “real world” of changing health risks, increasing health disparities, and newly developing health technologies (among other pressing challenges). Educational programs should have some degree of flexibility in specifying rotations at different health care services, so as to provide a more comprehensive practical experience of public health issues. In Italy, over the course of 4 years of training in Public Health, residents learn to apply and develop a set of skills under the guidance and mentorship of senior managers (Inter-ministerial decree 2015). Such rotations should provide these trainees with the opportunity to nurture their governance and leadership skills under expert supervision. Directors running such training programs may interpret these needs differently, however, and residents may sometimes receive no training at all on certain issues.

To provide a better understanding of the structured governance of health care systems during training programs, the first aim of this study was to assess public health residents’ perception of the training they had received on health system governance (HSG) and their perceived HSG resting training needs. The second aim of our work was to develop a competency framework for the “good governance” of a health system, which could guide the design of new Public Health physicians’ curricula. Our ultimate goal was to improve public health professionals’ ability to govern health services.

## Methods

### Context

The Italian healthcare system is essentially region-based and organized on three levels: national, regional, and local. Under the Italian Constitution, responsibility for health care is shared by the central government and 20 regional authorities (Constitution of Italian Republic 1948).

Only the former has the power to establish ‘essential levels of care’ or basic packages that must be available to all residents throughout the country, and it is responsible for ensuring that the general objectives and fundamental principles of the national health system are met. The regional authorities are virtually entirely responsible for organizing and administering public-financed health care. Responsibility for health care planning is shared by the central government and the regional authorities. Regional/local planning is based on national health plans, and regional authorities have to integrate national directives in their regional/local health plans (Ferré et al. 2014). Responsibility for health service delivery lies with the geographically distributed local health enterprises (Aziende Sanitarie Locali, ASLs), which are public-owned companies legally forming part of the regional authorities. The ASLs are responsible for assessing needs and providing comprehensive care to their local population, using either their own staff and facilities or contracting the services out to public hospital enterprises and for-profit and non-profit independent hospitals and specialist outpatient service providers. Private providers must be accredited and have a contract with the ASL. Tax contributions allocated to the National Health Fund have been redistributed horizontally between the regions using a weighted capitation mechanism. ASL services are financed by the regional governments. Each ASL is managed and governed by a general manager appointed by the regional department of health, based on his/her professional qualifications and technical skills. This general manager appoints a financial manager and a medical director. Services are structured according to a typical division-based model. Each division has financial autonomy over and technical responsibility for one of three different health care system areas (Legislative decrees No. 502/1992 and No. 229/1999):

- directly managed acute care and rehabilitation hospitals provide hospital-based acute inpatient, outpatient, and rehabilitation care;
- health districts are sub-ASL geographical units responsible for coordinating and providing primary care, non-hospital-based specialist medicine, and residential and semi-residential care to their assigned populations; district physicians provide home care services, drug abuse prevention services, and care for terminal AIDS patients;
- health promotion divisions are responsible for promoting health and preventing infectious and other diseases in the community; these divisions also provide services for monitoring environmental hazards, preventing occupational injuries, and supervising the production, distribution and consumption of food and beverages.
- ASL divisions are organized as departments and run by healthcare professionals, who must have the clinical and managerial expertise needed to provide the health services, design an annual plan of activities, and allocate the resources obtained by means of negotiations with the ASL’s general manager, consistently with regional and local plans and service programs. A department’s activities are programmed, implemented, and monitored with the active participation of health professionals assigned to the department.

### Survey design

The first part of our research involved a cross-sectional study conducted between March and September 2011, during which an anonymous questionnaire was administered to all Italian medical residents on postgraduate courses in Hygiene and Preventive Medicine. The questionnaire was designed to assess public health residents’ perception of the training they had received on HSG and their perceived HSG training needs. It was formulated by a scientific working group created by the Italian Association of Medical Managers (SIMM), established in 2006, and the Council of Hygiene and Preventive Medicine Trainees (Consulta degli Specializzandi SItI), established in 1998 as an agency of the Italian Scientific Society of Hygiene, Preventive Medicine and Public Health (SItI). In Italy, access to public health careers for medical doctors requires this specialty in most cases. Schools of Hygiene and Preventive Medicine are state-run and offer postgraduate training courses that correspond—according to the European Union’s system for the recognition of professional qualifications—to training courses in Community Medicine. The minimum duration of the course is 4 years (Council Directive 2006/100/CE EC). The majority of Public Health medical doctors are employed in primary care, health promotion divisions, hospital management units, and regional or national public health agencies.

The questionnaire was sent by e-mail; it consisted of 33 questions, with a brief socio-demographic section and three parts concerning the following domains: (1) work experience gained in some key HSG areas during the entire period of training; (2) perceived need for training in HSG areas. Answers were given on a 5-point Likert scale for training received (where 1 meant ‘never’, and 5 meant ‘most of the time’), and for perceived training needs (where 1 = very low, and 5 = very high). An appropriate descriptive analysis was conducted, and absolute and relative frequencies were reported for categorical variables.

### Health system governance framework design

The second aim of the project, after describing the present situation in Italy as concerns training on HSG issues, with a view to helping directors of courses of specialization in hygiene and preventive medicine to cover all the competences required in HSG, was to define what the “good governance” of a public health system entails, in terms of the roles and responsibilities involved. Our public health system governance framework was developed based on the roles and responsibilities at various levels of governance in the context of the Italian health system. In defining roles and responsibilities, we considered ‘planning’ with two different functions: strategic planning and operational programming.

For each role, we identified the basic functions of the public health system’s steward-managers, considering their respective positions in the ASL’s divisional model and the activities required of each function. Competence integrates multiple components such as knowledge, skills, values and attitudes and changes with time, experience, and setting (Frank et al. 2010). We defined the competences needed for the various activities. We then generated a list of governance competencies that public health professionals need to have acquired in relation to their position within the health system, competencies that need to be learned by means of courses of specialization in public health, hygiene and preventive medicine, and higher-level continuing professional development schemes.

## Results

Twenty-five (78.1 %) of the 32 Italian postgraduate schools of public health took part in this survey, and 299 (55.9 %) of the 535 public health residents attending courses in 2011 completed the questionnaire. Respondents were of a mean age of 32.4 ± 4.5 years (median 31 years), and 65.9 % of the sample were females. Ninety-four residents (31.4 %) were attending the first year of the course, 41 (13.7 %) the second, 52 (17.4) the third, and 112 (37.5 %) the fourth.

Table 1 shows the percentage distributions (for each year of the course) of the public health issues never or almost never covered by the training that residents had received on HSG issues and that do not need further educational investments. The number of residents who had never or almost never received training on a given topic dropped over the 4 years of the course, but the topic of inequality-reducing strategies had never or almost never been covered by more than half of the residents, not even those in their last year of training. The questionnaire revealed that residents felt the need for more training on all the proposed HSG-related topics, and especially on mass media communication, health service programming, and human resource management.

**Table 1:** Postgraduate trainees’ perceived need for training on Health System Governance related topics, with percentage distributions for each year of the course (data extracted from responses to the questionnaire completed by Italian physicians attending courses on Hygiene and Preventive Medicine in 2011)

	I *n* = 94 (%)	II *n* = 41 (%)	III *n* = 52 (%)	IV *n* = 112 (%)	*P* value
Public health issues never or almost never covered by ongoing training experience					
Health service programming	55.43	24.39	32.69	25.89	<0.001
Mass media communication	50.00	36.59	26.92	35.71	0.037
Communication with policy-makers	60.87	46.34	40.38	44.64	0.053
Complex adaptive system management	47.83	34.15	19.23	24.11	<0.001
Leadership	57.61	36.59	38.46	38.39	0.019
Human resource management	53.26	41.46	42.31	33.04	0.037
Inequality-reducing strategies	57.61	41.46	51.92	50.89	0.386
Issues on which no further training is needed					
Health economics	7.61	2.44	7.69	16.07	0.046
Health service programming	6.52	4.88	0.00	13.39	0.018
Mass media communication	6.52	4.88	3.85	9.82	0.484
Communication with policy-makers	6.52	2.44	1.92	10.71	0.114
Leadership	5.43	4.88	3.85	17.86	0.004
Human resource management	6.52	2.44	3.85	12.50	0.091
Inequality-reducing strategies	6.52	9.76	7.69	21.43	0.006

**Fig. 1:**
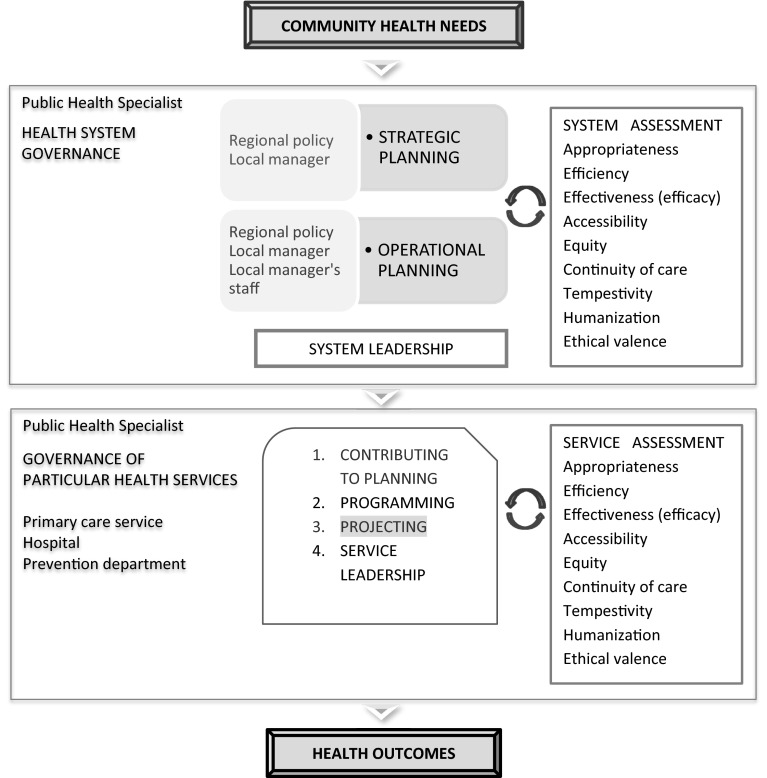
All-round planning model: roles in health system governance/health services governance (Italy 2015)

The competencies needed for a good health system governance, based on the roles and responsibilities of medical doctors working for the Italian National Health Service (NHS), were defined for each of the different levels of governance involved in this system. Two different governance functions, health system governance and health service governance, were considered when defining roles and responsibilities. The planning process consists of different stages and requires the following activities at each stage: situation analysis and priority setting, considering the options, programming, implementation, monitoring, and assessment. From the governance perspective, a health system should be assessed considering specific parameters: appropriateness, efficiency, efficacy and effectiveness, accessibility, equity, continuity of care, promptness, humanization, and ethical worth (Fig. 1).

Table 2 lists the subjects involved in health system governance: the Regional Health Directorate, the ASLs, and the managers and public health professionals who run a given health service. Tables 3, 4, and 5 show the activities and competences relating to each level of governance.

**Table 2:** Public health system governance: subjects, roles, functions (Italy, 2015)

Subjects	Roles	Functions
Regional health directorate, LHU (local health unit) manager	Strategic planning**—**regional and local levels	Defining principles/values Assessing health needs Establishing priority goals/health policies
	Operational planning—local level	Establishing strategic objectives and initiatives
	System leadership role—local level	Devising policies and goals in cooperation with the Regional health department Negotiating budget/extras tailored to achieve objectives Coordinating health services Approving budget and monitoring expenditure Managing community relationships Managing relations with private accredited providers (based on regional and local health goals) Managing personnel and other human resources Managing support services Managing communications
Governance of particular health services		
Level I subjects: structure manager, e.g. of acute care and rehabilitation hospitals, health districts, health promotions	Cooperating on planning Service programming Service planning Service leadership role	Negotiating goals for services Defining and monitoring services programs Defining and monitoring operational services projects Resource programming Cooperating with other health care structures Personnel management Process management Cost management
Level II subjects: health service manager, e.g. as head of hospital or district, or prevention service	Cooperating on programming and planning	Designing and monitoring operational projects
	Health service leadership role	Cooperating with other services
	Work management	Personnel management Process management Cost management Assessing production processes

**Table 3:** Subjects: Regional Health Directorate, ASL (local health enterprise) manager (Italy 2015)

Function	Activities	Competency
Defining principles/values	Adopt National Health System principles Community values assessment Define the principles for organizing health care providers and for providing health care services	Understand NHS principles and current public health issues, and engage in systemic changes to address them Understand relevant but competing values that are perceived to be legitimate and fair Understand relevance to the local setting, decided on the strength of shared criteria; publicize priority-setting decisions and the reasons behind them Provide leadership to ensure that the above conditions are met
Assessing health needs Establishing priority goals/health policies	Identify expressed and unexpressed needs	Use epidemiological, qualitative, and comparative methods to describe the health problems of a population^a^ Identify inequalities in health service provision and access Establish priorities for the most effective use of resources Understand the benefits achievable with health care or wider social and environmental changes Have a practical understanding of what is involved, the time and resources needed to undertake assessments, and ensure sufficient integration of the results in the planning and commissioning of local services
	Identify the importance of a need in terms of frequency or severity, the evidence of the efficacy of interventions, or the feasibility of change (from social, health and economic perspectives) Define criteria coherent with principles to prioritize needs Involve other organizations such as social services, local authorities, volunteer groups, etc.… in the process of defining priorities	
	Defining health objectives	Establish priorities/value-laden choices/address a broader range of relevant values, such as trust, equity, accountability and fairness that are of concern to other partners and, not least, to the populations concerned Model effective group process behavior including listening, exchanging opinions, negotiating, rewarding, encouraging, and motivating
Establishing strategic objectives and initiatives	Analyze relationships between needs, and the supply and demand of services, to ascertain met and unmet needs Involve other organizations such as social services, local authorities, volunteer groups, etc.… (adopting principles and priorities defined by strategic planners) Establish strategic objectives and initiatives, together with financial and organizational criteria for managing health care organizations (based on objectives and principles defined by strategic planners)	Epidemiological and economic analysis: conduct the analysis in the same way as for any good or service, but bear in mind that it has peculiar features that may mean that the usual assumptions on the effects of markets on resource allocation do not hold Epidemiological and economic analysis: judge the degree to which scarce resources are employed according to two main criteria: efficiency and equity Translate broad strategies into practical terms for others; build alliances, partnerships, and coalitions to improve the health of the community or population being served; identify and engage stakeholders in interdisciplinary projects to improve public health Monitor efficiency, effectiveness and appropriateness Understand the attribution or contribution of observed results to a program; define new policies and programs that respond to these challenges
	Program assessment	Design assessments so that they reflect a program’s stage of development, define the purpose of the assessment, its uses, and the questions to be answered
Devising policies and goals in cooperation with the regional health department	Legislation provides the general manager with substantial autonomy in managing human, financial and technological resources. This autonomy is expressed in: A three-year strategic plan in which the general manager defines the organization’s mission and goals, which should be consistent with the regional health plan An annual strategic program in which the general manager defines annual projects that contribute to achieving the objective in the plan	Make strategic decisions based on recognized values, priorities and resources Identify and communicate new system structures as needs are identified and opportunities arise Ensure that organizational practices are aligned with changes in the public health system and the larger social, political, and economic environment
Negotiating budget/extras tailored to achieve objectives	Negotiate global budget (ASL services are financed under a global budget) Negotiate additional funding (for purchasing technologies/building works/…)	Demonstrate the ability to interpret and explain financial and managerial accounting information, prepare and analyze budgets, and make sound financial management decisions
Coordinating health services	Define objectives for health services Ensure that financial resources are committed to those activities that contribute to organizational goals (budget allocation for health services) Develop and manage shared projects (define objectives, support for and supervision of Functional Departments)	Enable health services to create, communicate and apply shared visions, missions and values Contribute to developing key values and a shared vision in planning and implementing programs Build on capacity: improve performance and enhance the quality of the working environment Contribute to maintaining the organizational performance standards of health services Demonstrate an ability to build on the capacity of health services by sharing knowledge, tools, expertise and experience and resources Enable health services to share a patient-centered vision of health care
	Program assessment	Demonstrate the ability to monitor the program’s stages of development, and to identify and remove obstacles to the achievement of the objectives
Approving budget and monitoring expenditure	Re-direct resources, reorganize health services to maximize the impact of healthcare on health needs Make carefully considered strategic decisions to ensure that health services are stable and sustainable into the future Financial management and cost control	Assess the costs and benefits of shifting resources, Identify different expenditure patterns, explain them to the population at large, and engage the public more meaningfully in decisions on future spending patterns. Demonstrate the ability to draw up plans for outlays and incomes based on history and an understanding of what the future holds Make decisions on resource allocation, and contribute to the effectiveness of the health care system
Managing community relationships	Manage relations with politicians Manage relations with citizens	Create and communicate a shared vision for the future and inspire the community to achieve it Translate broad strategies into practical terms for others Build alliances, partnerships, and coalitions to improve the health of the community or population being served Identify and engage stakeholders in interdisciplinary projects to improve public health Advocate and participate in public health policy initiatives at local, national and/or international levels
Managing relations with private accredited providers (based on regional and local health goals)	Define types of service to have supplied by private accredited providers on the grounds of unmet needs Negotiate the volume of services and financing with private providers Systematically monitor the volume and quality of services supplied by private providers	Demonstrate the ability to negotiate with private accredited providers for services to be supplied
Managing personnel and other human resources	Conduct job analyses Plan personnel needs and recruitment Select the right people for the job Career orientation and training Provide benefits and incentives Assess performance Solve disputes Communicate with all employees at all levels	Advocate for learning opportunities within the organization Create and communicate a shared vision for the future and inspire team members to achieve it Encourage others to have a sense of ownership of the public health mission in the organization Help others to clarify their ideas, create consensus, and develop ideas into practicable plans Offer opportunities for collaborative learning and quality improvement Develop and mentor potential future leaders within the organization
Managing support services	Define aims and tasks for support services Monitor and assess support services Plan, monitor, and assess outsourcing	Create and communicate shared aims for health and support services Engage support services in interdisciplinary projects to improve public health Identify activities to outsource and the entity of these activities; communicate the reasons for outsourcing them
Managing communications	Health communication Risk communication	Demonstrate the ability to develop communication strategies, design internal and external communication directives, and manage the flow of information Demonstrate the ability to ensure that the logic, outcomes, significance, and limitations of the risk assessment are clearly understood by all stakeholders

^a^ Core Competencies for Public Health Professionals - Analytical/Assessment Skills. Council on Linkage Between Academia and Publich Health Practice (2014)

**Table 4:** Governance of particular health services–Level I: Subjects: Structure Manager (Italy,2015), e.g. Managers of acute care and rehabilitation hospitals—Managers of health districts

Function	Activities	Competency
Negotiating goals for services	Adopt general health objectives (based on regional and local planning) Propose health objectives, based on regional and local values and principles, for future planning in order to deal with unmet needs	Use evidence- and research-based health policies and programs
Defining and monitoring service programs	Propose program objectives consistent with regional and local plans Give local managers feedback on the progress of the program (timetable/resources/intermediate and final objectives)	Analyze information to identify appropriate implications, uses, gaps and limitations Establish the meaning of information, considering the current ethical, political, scientific, socio-cultural and economic contexts Recommend specific actions based on the analysis of information
Defining and monitoring operational service projects	Suggest project objectives consistent with regional and local plans and service programs Define operational objectives Define strategies/timetables/resources/intermediate and final objectives Define guidelines and protocols Define outcome and process indicators Assess operational projects Give local managers feedback on the progress of projects (timetable/resources/intermediate and final objectives)	Describe program options chosen to address a specific public health issue Describe the implications of each option Develop a program for taking action bearing in mind relevant evidence, legislation, emergency planning procedures, regulations and policies Implement a program to address a specific public health issue Demonstrate the ability to coordinate the process for implementing good practice guidelines Assess an action or program Demonstrate the ability to establish and follow priorities, and to maximize outcomes based on available resources
Resource programming	Suggest budget objectives consistent with service objectives to local managers	Demonstrate the ability to translate health objectives into service objectives
Promoting personnel to achieve defined objectives	Negotiate training aims with personnel Propose training consistent with objectives of regional and local plans and service program	Be aware that the staff is the most valuable asset in any business
Cooperating with other services	Cooperate with local manager and other services on the management of shared actions Cooperate with support services	Cooperate to optimize performance by sharing resources and responsibilities Use skills such as team building, negotiation, conflict management and group facilitation to build partnerships
Personnel management	Assess the need for human resources to achieve service goals Assess the skills of human resources Allocate human resources Guarantee personnel safety Define individual and group objectives for personnel Assess personnel based on their achievement of individual and group objectives Cooperate with local manager and personnel office	Describe the mission and priorities of the public health organization where you work, and apply them in practice Contribute to developing key values and a shared vision in planning and implementing public health programs Contribute to team and organizational learning in order to advance public health goals Demonstrate the ability to build team spirit by sharing knowledge, tools, expertise and experience
Process management	Process monitoring: define process indicators Adopt surveillance systems (care associated infections, clinical risks) Quality/efficacy/patient safety monitoring: define outcome indicators	Contribute to maintaining organizational performance standards
Cost management	Monitor the achievement of budget objectives Monitor the use of resources	Demonstrate the ability to manage resources

**Table 5:** Governance of particular health services—level II: health service manager (Italy 2015), e.g. Head of a hospital or health district, or prevention service

Function	Activities	Competency
Designing and monitoring operational projects	Adopt objectives of local plans and service programs and propose operational programs to Department Directors Propose operational objectives Propose specific strategies/processes/timetable/resources needed Propose guidelines and protocols Propose outcome and process indicators Collect data for operational project assessments Give Department Director feedback on progress of operational projects (timetable/resources/intermediate and final objectives)	Develop projects for taking action bearing in mind relevant evidence, legislation, emergency planning procedures, regulations and policies Implement a project and/or take appropriate action to address a specific public health issue Demonstrate the ability to implement good practice guidelines Demonstrate the ability to keep to priorities, and to maximize outcomes based on available resources
Cooperating with other services	Cooperate with other services and units on the management of shared actions	Cooperate to optimize performance by sharing resources and responsibilities
Personnel management	Assess the need for human resources to achieve the unit’s goals Assign personnel tasks and responsibilities Ensure personnel safety Define individual and group objectives for personnel Assess personnel based on their achievement of individual and group objectives Manage available spaces and working patterns	Recognize staff’s talents and skills Give personnel opportunities to learn and do new things Give personnel feedback on their performance to help them improve their performance and enhance work efficiency Use skills such as team building, negotiation, conflict management and group facilitation Understand and adopt principles and of appropriateness, efficiency, humanization, equity… Understand the needs of individuals, the needs of teams, the needs of the organization/patient groups
Process management	Define production processes consistent with objectives and in accordance with principles of appropriateness, efficiency, humanization, equity… Monitor budget objectives related to process management	Implement, coordinate and monitor production processes to address specific service objectives
Cost management	Monitor usage of resources	Demonstrate the ability to manage resources
Assessing production processes	Give Department Director data for use in process assessments Discuss the achievement of objectives with personnel and identify areas needing improvement	Assess an action or program Analyze information to identify appropriate implications, uses, gaps and limitations

## Discussion

The findings of this study show that not all HSG-related topics were adequately covered during the 4 years of postgraduate training received by our sample. On the whole, the trainees reported feeling the need for more training on all the broad topics considered, and especially on health service organization, human resource management, health communication and advocacy, and communication with policy-makers (and this was still true, albeit to a lesser degree, for trainees nearing the end of their course). Judging from our results, even though the proportion of residents reporting that they had received training of advocacy and communication with policy-makers increased over the years, more than 40 % of the residents in the last year of their training had still gained no experience of such matters. In their relations with the public health operators of tomorrow, policy-makers may have more than a passive role as recipients of the latter’s communications. Education for public health operators should be provided within the academic and professional spheres, of course, but policy-makers should be involved too. Public health skills and concepts need to be disseminated and engage more with local communities and policy-makers (Gebbieet al. 2003).

Another point of interest concerns the lack of incremental training on issues related to the adoption of strategies to reduce healthcare disparities during postgraduate training courses. This is an enormous shortcoming for any training on matters of HSG, bearing in mind that the 2020 vision for the education of our next generation of public health leaders recommends that all students be imbued with the fundamental value of the relationship between individual and community health (Koh et al. 2011). Public Health professionals have to consider social determinants in all aspects of their work with a view to achieving the far-reaching goals of equity in healthcare, eliminating disparities and improving the health of all social groups.

A population healthcare-oriented approach should be promoted when defining medical education programs to shape future public health professionals capable of serving as stewards and managing the system’s resources (Gray 2013).

The need for a better education on all HSG-related topics reported by trainees in the last year of their postgraduate course could be interpreted as a failing of the schools of specialization. Our residents’ perceived need for further education could, therefore, be seen in a less negative light and interpreted as an appropriate demand for continuing professional development. The US Institute of Medicine of US Academies states that education in the public health sector—management aspects included—should aim to instill a culture of lifelong learning (Gebbie et al. 2003). The approach taken to public health professionals’ education can go beyond the traditional task of transmitting information to the more challenging job of developing the skills needed to access, discriminate, analyze, and use knowledge (Gebbie et al. 2003). Now, more than ever, the duty of academic institutions is to teach students how to think creatively to master large flows of information in search of solutions.

This survey can serve as a starting point for future research with a view to designing a training plan for public health professionals that is better tailored to local priorities. A crucial gap exists between current public health needs and the extent to which public health workers are trained during their basic health education (Paccaud et al. 2013). Berkenbosch et al. (2013) showed differences in the perceived educational needs of medical residents in various countries that were attributable to the different structure of the health care systems in which they worked. Paccaud et al. (2013) suggested that, to be better able to understand the needs of the workers, and to prepare our educational institutions to meet these needs, we need a more cohesive understanding of where public health graduates are going when they enter the workforce and what they are doing. Public health academics need to develop training plans that respond to the needs and priorities of sometimes very diverse local contexts.

Bjegovic-Mikanovic et al. (2013) found that public health education in Europe suffers from deficits in modernity, especially as regards continuing education, and the size and total number of public health schools and departments in Europe is far from being sufficient when compared with the levels achieved in the US. Public health schools and departments need to increase their efforts to ensure continuing professional development by designing and delivering training courses tailored to the needs of public health professionals and employers (Vukovic et al. 2014).

Our survey did not assess the ability of public health medical employees to do their jobs, but we did define a framework of competences needed for good governance, with a view to highlighting the skills that public health professionals in stewardship roles on various levels have to use to govern the services of a public health system. Each level of training, specialization, Masters, PhD courses or similar schemes, has to assure the acquisition of competencies suited to the roles and functions of the various public health professionals and employers.

The method of representing competences in tiers was also adopted by the Council on Linkages between Academia and Public Health Practice (2014) in a document that identifies three levels of public health professional: Front Line Staff/Entry Level, Program Management/Supervisory Level, and Senior Management/Executive Level. Only two of these levels have managerial roles, however, whereas our framework included some managerial skills even for the first level in the hierarchy.

Public health starts with a foundation of science but inevitably requires moving into the dynamic realms of social strategy, political will, and interpersonal skill (Koh et al. 2011). Public health schools and departments need to develop more progressive curricula (Czabanowska et al. 2014b). In order to gain knowledge and skills, both basic and specific training objectives in the academic setting and professional training activities in health services are required. New training strategies must also be considered such as team-based learning (Frenk et al. 2010) and blended learning (Czabanowska et al. 2014b). Finally, young professional networks could offer their members to develop competencies in a multidisciplinary and international contest (Boyle and Ribeiro 2014).

No previously published studies appear to have defined such a framework of competencies needed for good governance, though the concept of good governance has attracted a good deal of attention in the public administration sector. In ‘New Public Management’, good governance means working together coherently without relying on governments, but rather following a principle of subsidiarity. The concept of good governance is increasingly being discussed in the context of public health too (Boffin 2002). Good governance is both ethically essential and instrumental to public health (Mikkelsen-Lopez et al. 2011). Good governance is part of a national-scale effort to achieve well-being through a ‘Health in all Policies’ approach, for instance, focusing all stakeholders on the target of improving population health; but it should be a core concept for public health on a local level too (Brand 2007).

This study tried to overcome the lack of consensus on the dimensions of governance, their scope, and purpose, by identifying the governance functions related to the tasks and responsibilities of public health professionals in stewardship roles. These governance functions were divided into levels. On the higher level of health system governance (strategic and operational planning activities), we have the Regional Health Directorate and the ASL manager. There are two different levels involved in operational programming, i.e. structure managers (hospitals manager, health districts manager, health promotion manager) and managers of a particular health service (a head of an hospital, or district or prevention service). Our study suggests that the core competencies needed for good governance could be taught sequentially, progressing from the lower to the higher stewardship roles.

The competences described by the previously mentioned Council on Linkages Between Academia and Public Health Practice (2014) are organized into eight domains, not functions, and they form a consensus set of skills for the broad practice of public health in any health system.

Many other competency frameworks have been developed for the public health sector and there is a fairly strong consensus in the USA and Europe on what key competency areas should be included in academic public health curricula (Czabanowska et al. 2014a). As part of the Leaders for European Public Health (LEPHIE) Erasmus Multilateral Curriculum Development Project supported by the EU Lifelong Learning Programme, a public health leadership competency framework was recently outlined to support the development of a competency-based European public health leadership curriculum (Czabanowska et al. 2013, 2014a).

The framework described here was designed to respond to the need for clarity and consensus regarding a set of essential competencies reflected in the practice of stewardship. It could also be useful for planning professional training in both the academic and the health system settings (for postgraduate training and continuing professional development). Today’s public health educational institutions and public health professionals are searching for an interface and synergies between science and practice. Our framework could also be useful for assessing the curricula of public health professionals wishing to be employed in stewardship roles.

This study is affected by several limitations. The questionnaire administered did not assess all areas of training; nevertheless, the results indicated that some fundamental areas were not covered suggesting the need for a better structured HSG framework of competence to accomplish during the training period.

### Conclusion

Our survey among Italian public health residents revealed a significant gap between the training currently provided for public health professionals and what these trainees would have wished to receive, in view of their future role and functions in our public health system. We defined the competencies needed for good governance in the setting of public health professionals operating in stewardship roles. The framework of competencies proposed here is by no means intended as an end product. Instead, it is offered to public health system experts as a preliminary tool for them to assess, test, revise, and improve. Good governance in public health systems is the ultimate goal and action must be taken to work towards this ideal.
